# Evaluation of droplet digital PCR rapid detection method and precise diagnosis and treatment for suspected sepsis (PROGRESS): a study protocol for a multi-center pragmatic randomized controlled trial

**DOI:** 10.1186/s12879-022-07557-2

**Published:** 2022-07-19

**Authors:** Yuanhan Zhao, Ke Lin, Haocheng Zhang, Guanmin Yuan, Yanliang Zhang, Jingye Pan, Liang Hong, Yan Huang, Ying Ye, Lisu Huang, Xiaohua Chen, Jun Liu, Xiang Li, Xiaoju He, Qiaoyan Yue, Hong Zhang, Aiming Zhou, Yangyang Zhuang, Jie Chen, Caixia Wu, Wei Zhou, Fujing Cai, Shengguo Zhang, Liang Li, Shaling Li, Tingting Bian, Jiabin Li, Jun Yin, Zhengshang Ruan, Shanshan Xu, Yan Zhang, Jie Chen, Ying Zhang, Jun Han, Tingting Su, Fan Tu, Lijing Jiang, Chen Lei, Qiu Du, Jingwen Ai, Wenhong Zhang

**Affiliations:** 1 Department of Infectious Diseases, National Medical Center for Infectious Diseases, Shanghai Key Laboratory of Infectious Diseases and Biosafety Emergency Response, Huashan Hospital, Fudan University, Shanghai, China; 2 Department of Infectious Diseases, The Nanjing Hospital of Chinese Medicine, Affiliated to Nanjing University of Chinese Medicine, Nanjing, Jiangsu China; 3 Department of Intensive Care Unit, The First Affiliated Hospital of Wenzhou Medical University, Wenzhou, Zhejiang China; 4 Department of Infectious Diseases, Ruian People’s Hospital, Ruian, 325200 China; 5 Department of Infectious Diseases, Xiangya Hospital Central South University, No. 87 Xiangya Road, Changsha, 410000 Hunan China; 6 Department of Infectious Diseases, The First Affiliated Hospital of Anhui Medical University, Hefei, Anhui China; 7 Department of Infectious Diseases, Xinhua Children’s Hospital, Xinhua Hospital, Shanghai Jiao Tong University School of Medicine, Shanghai, China; 8 Department of Anesthesiology and Surgical Intensive Care Unit, Xinhua Hospital Affiliated to Shanghai Jiao Tong University, School of Medicine, Shanghai, 200092 China; 9 Department of Infectious Diseases, Shanghai Sixth Hospital, Shanghai Jiaotong University, Shanghai, China; 10 Department of Laboratory, Wuxi No. 5 People’s Hospital Affiliated to Nantong University, Wuxi, Jiangsu China; 11 Department of Infectious Diseases, Wuxi No. 5 People’s Hospital, Wuxi, Jiangsu China; 12 Department of Critical Care Medicine, Minhang Hospital, Fudan University, No. 39, Xinling Road, Minhang District, Shanghai, 201199 China; 13 Department of Pharmacy, The Nanjing Hospital of Chinese Medicine Affiliated to Nanjing University of Chinese Medicine, Nanjing, Jiangsu China; 14 National Clinical Research Center for Aging and Medicine, Huashan Hospital, Fudan University, Shanghai, China; 15 Key Laboratory of Medical Molecular Virology (MOE/MOH), Shanghai Medical College, Fudan University, Shanghai, China

**Keywords:** Sepsis, Septic shock, Multicenter, Randomized trial, Rapid diagnostics

## Abstract

**Background:**

Sepsis is still a major public health concern and a medical emergency due to its high morbidity and mortality. Accurate and timely etiology diagnosis is crucial for sepsis management. As an emerging rapid and sensitive pathogen detection tool, digital droplet PCR (ddPCR) has shown promising potential in rapid identification of pathogens and antimicrobial resistance genes. However, the diagnostic value and clinical impact of ddPCR tests remains to be studied in patients with suspected sepsis. PROGRESS trial is aimed to evaluate the clinical effectiveness of a novel ddPCR assay compared with standard practice.

**Methods:**

PROGRESS is a multicenter, open-label, pragmatic randomized controlled trial (pRCT) set in ten hospitals, including departments of infectious disease and intensive care units. In this study, a total of 2292 patients with suspected sepsis will be randomly assigned to two arms: the ddPCR group and the control group with a ratio of 3:1. The primary outcome is the diagnostic efficacy, that is, the sensitivity and specificity of the ddPCR assay compared with the synchronous blood culture. Secondary outcomes include the mortality rates and the mean Sequential Organ Failure Assessment (SOFA) score at follow-up time points, the length of stay in the hospital, the time to directed antimicrobial therapy, duration of broad-spectrum antibiotic use, and the EQ-5D-5L score on day 90.

**Discussion:**

It is the first multicenter pragmatic RCT to explore the diagnostic efficacy and clinical impact of the ddPCR assay in patients with suspected sepsis, taking advantage of both RCT’s ability to establish causality and the feasibility of pragmatic approaches in real-world studies (RWS). This trial will help us to get a comprehensive view of the assay’s capacity for precise diagnosis and treatment of sepsis. It has the potential to monitor the pathogen load change and to guide the antimicrobial therapy, making a beneficial impact on the prognosis of sepsis patients.

*Trial registration*: ClinicalTrial.gov, NCT05190861. Registered January 13, 2022—‘Retrospectively registered’, https://clinicaltrials.gov/ct2/show/NCT05190861.

## Background

Sepsis is life-threatening organ dysfunction caused by a dysregulated host response to infection [1]. It is considered a significant health concern worldwide for its high morbidity and mortality, which is between one in three and one in six [2]. As a medical emergency, early identification and management are crucial to better clinical outcomes. According to the 2021 Surviving Sepsis Campaign (SSC) guidelines, there is a strong recommendation for administering antimicrobials immediately, ideally within one hour for adults with possible septic shock or a high likelihood for sepsis [2]. The delayed initiation of effective antimicrobial therapy results in worsened outcomes [3], antibiotic misuse problems and a heavier health economics burden.

In clinical practice, physicians count on the information of the causative pathogens to start a precise antimicrobial therapy, highlighting the need for rapid and accurate pathogen identification tools. Currently, blood culture (BC) with antimicrobial susceptibility testing (AST) is the gold standard for diagnosing bloodstream infections (BSIs). However, this standard management is limited by a rather longer turnaround time (TAT). It takes around one to three days for the organisms, sometimes 28 days for the tuberculosis, to be cultured on the specific medium and additional one to two days to provide AST results [4, 5]. The sensitivity of the blood culture is relatively low and it can sometimes give false-positive results because of contamination [4, 6].

In addition to optimizing the culture procedure and inventing methods applied to positive blood culture bottles, emerging molecular rapid diagnosis tests (mRDTs) directly using whole blood samples are currently in the ascendant. These methods can be divided into two categories based on the technical principle: the sequencing techniques and the nucleic acid amplification (NAA) techniques. The former, such as metagenomic next-generation sequencing (mNGS) and nanopore sequencing, are capable of providing unbiased pathogen detection, while the latter specifically target and amplify nuclear acid sequence [7]. Most commercialized NAA methods like SeptiFast and Magicplex are based on real-time quantitative PCR in combination with other techniques, and have been able to shorten the TAT to 6–10 h with less hands-on time [6, 8]. However, some have been withdrawn from the market because of the low sensitivity, underlining the challenge in investigation and implementing of such mRDTs [9, 10].

Among the NAA techniques, droplet digital PCR (ddPCR) stands out for its high sensitivity, the ddPCR also has the capability of absolute nucleic acid quantification by counting the number of positive/negative droplets at the end of PCR reaction and applying Poisson statistical analysis without need of external references. It has been applied to quite a lot fields including liquid biopsy and non-invasive prenatal testing. Recently the ddPCR based assays have also shown promising potential in microbial infection for its ability to detect pathogens at a low amount of plasma DNA fragments which is estimated to be as few as 10 colony-forming unit (CFU) per milliliter in about 4 h’ TAT [11–13]. Therefore, the etiological test result could be obtained on the same day as sepsis was first suspected.

There have been many studies exploring the diagnostic efficacy of novel mRDTs in patients with suspected sepsis, and several of the very ddPCR based assays. However, most of them are single-centered observational studies without evaluating clinical impact nor cost effectiveness of such tests [9, 11, 14]. And the proportion of disagreement with the reference test varied across studies, indicating that a huge impediment to sepsis research is the poor homogeneity, which partially explains why many large RCT studies failed to improve the prognosis of sepsis patients [15–18]. Therefore, there is lack of clinical evidence supporting the value of the novel mRDTs currently, so the potential clinical benefits and risks need to be assessed in real world. This trial aims to evaluate the diagnostic efficacy of the ddPCR assay, examine its consistency with conventional BC results, and further exploring the clinical impact and application value in patients with suspected sepsis.

## Methods

### General study design

**Fig. 1:**
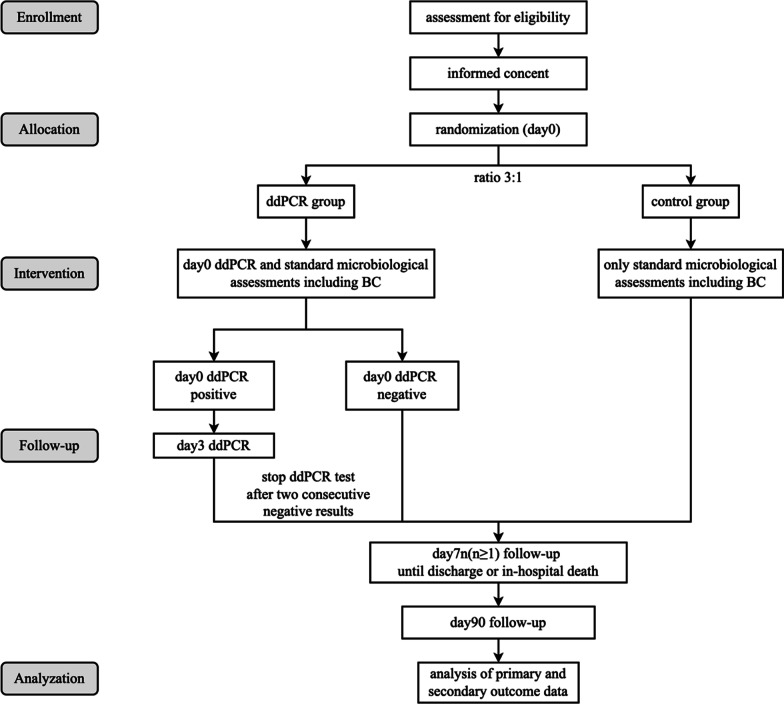
Flowchart of PROGRESS trial procedure

This trial is a multicenter, open-label randomized control trial recruiting patients with suspected sepsis. Eligible patients will be included after signing the written informed consents. Then, the enrolled patients will be allocated into two parallel arms using stratified block randomization method with a ratio of 3:1. The two groups of participants are those whose pathogen detection will be achieved through both the ddPCR assay and standard microbiological assessments including a synchronous blood culture (experimental group/ ddPCR group), and those being assessed only through standard microbiological assessments including blood culture and other achievable clinical diagnostic methods like mNGS (comparative group/control group). The detailed study flowchart is shown in Fig. 1.

### Setting

This multi-center trial is set in the infectious disease departments and ICUs of ten hospitals, which are distributed across five provinces in China: Huashan Hospital Affiliated to Fudan University, Central Hospital of Minhang District, Xinhua Hospital Affiliated to Shanghai Jiao Tong University School of Medicine, and Shanghai Sixth People’s Hospital Affiliated to Shanghai Jiao Tong University School of Medicine in Shanghai; Nanjing Hospital of Chinese Medicine Affiliated to Nanjing University of Chinese Medicine and Wuxi No. 5 People’s Hospital in Jiangsu Province; Ruian People’s Hospital and The First Affiliated Hospital of Wenzhou Medical University in Zhejiang Province; Xiangya Hospital Central South University in Hunan Province; The First Affiliated Hospital of Anhui Medical University in Anhui Province. The Huashan PROGRESS project team is the trial sponsor and is responsible for the design and supervision throughout this trial.

### Study population

Adult patients who meet the inclusion criteria and none of the exclusion criteria will be eligible to participate in this trial (Table 1). They will be enrolled after providing the informed consent to their physician and the Huashan PROGRESS project team.

**Table 1:** Inclusion criteria and exclusion criteria

Inclusion criteria: 1. 18 years old or older 2. Meet at least two of SIRS^a^ criteria: ➀ Temperature > 38 ℃ or < 36 ℃ ➁ Heart rate > 90 bpm ➂ Respiratory rate > 20 or PaCO_2_ < 32 mmHg ➃ WBC > 12,000/µL or < 4000/µL or > 10% bands 3. Hospitalized patients who have a diagnostic blood culture drawn on the same day as enrollment (day 0), which is the standard procedure for suspected sepsis 4. Written informed consent signed by the patient or their legal representative Exclusion criteria: 1. Refusal to participate in the study or failure to comply with treatment or follow-up 2. Known pregnancy or lactation 3. The researcher believes that there are any conditions (social or medical) that allow subjects to participate is unsafe. For example, severe anemia or high risk of bleeding, etc., which are not suitable for taking peripheral blood for testing 4. Participating in other clinical studies	

*SIRS*^a^ system inflammatory reaction syndrome

### Randomization

After being enrolled in the trial, participants will be assigned randomly to either the ddPCR group or the control group using the stratified block randomization method. Stratification is designed to balance the two arms for age (< 65 years or ≥ 65 years) and setting (10 medical centers), the two factors that may jeopardize study conclusions. An online random system is built by a third-party company. The system is used to generate and save the random lists by giving separate blocks for each of the twenty combinations in advance. The block width is an integer multiple of 4 since the ratio is 3:1. In order to prevent manipulation of group assignment during recruitment, the specific block size will be disclosed at the end of this trial by the third-party company to the Huashan PROGRESS project team, who generates the allocation sequence after enrollment. Patients will be recruited consecutively and it is competitive enrollment among the medical centers.

### Blinding

There is no blinding in this trial. After randomization, the group information with corresponding procedure is open to the participants, their physicians and the project team. The Huashan PROGRESS project team will have access to the final trial dataset, and investigators from cooperative hospitals will have access to the data from their respective hospitals.

### Diagnostic methods

The ddPCR assay can identify over fifteen pathogens, covering the most common bacteria and fungi in BSIs according to the China Antimicrobial Surveillance Network 2021 Report (CHINET, http://www.chinets.com) [19] and seven antibiotic resistance genes. Droplet digital PCR analysis was performed using a 5-fluorescent-channel droplet digital PCR system (Pilot Gene Technology (Hangzhou) Co., Ltd.).

When evaluating the diagnostic test accuracy of ddPCR method, positive and negative results compared with the reference method (a synchronous blood culture) are defined according to a previous study [20]. The four possible results of the paired tests are in the following: Positive consistency (true positive, TP): In addition to the complete consistency between ddPCR results and the synchronous blood culture results, it also includes three situations: ddPCR results include additional pathogens, ddPCR test and simultaneous blood culture have results in common and both have independent detection of pathogens, ddPCR results are included in the simultaneous blood culture results; Negative inconsistency (false positive, FP): When the synchronized blood culture is negative, the ddPCR assay independently detects pathogens; Positive inconsistency (false negative, FN): When the ddPCR test is negative, the pathogens are independently detected by the synchronous blood culture. Or pathogens are independently detected by the ddPCR test and the synchronous blood culture without overlapping; Negative consistency (true negative, TN): No pathogen is detected by the ddPCR test nor the synchronous blood culture.

### Duration of follow-up

On the same day as randomization, which is counted as day 0, 10 mL peripheral blood samples will be collected. For the ddPCR group, blood samples will be sent for ddPCR tests and a synchronous blood sample will be collected for blood culture. Among them, the patients with positive ddPCR results for the first time on day 0 will have blood collected for monitoring on day 3, day 7, and day 7n(n > 1, that is, every seven days, until there is a clinical outcome). The patients with negative results for the first time on day 0 will have blood collected every seven days (on day7n, n ≥ 1). And if necessary, physicians can apply for an additional test for them at any time after day 3. For the control group, blood samples will be collected on day 0, and stored in the laboratories of participating institutions without applying the ddPCR test. The blood samples can be used for other microbiological tests such as mNGS, and any other microbial examinations are accessible to both the experiment group and the control group.

Apart from tests to identify pathogens, follow-up examinations also include laboratory examinations and physical examination results to calculate the severity of disease scores including SOFA and disseminated intravascular coagulation (DIC) scores. These are results of routine blood tests, serum C-reactive protein (CRP), procalcitonin (PCT), erythrocyte sedimentation rate (ESR), D-Dimer, arterial blood gas tests, blood culture, body temperature, respiratory rate, blood pressure, heart rate, state of consciousness and so on. Demographic information will be collected when patients are enrolled, and prognosis information when there is a patient outcome such as discharge or in-hospital death. Patients will also be phoned on day 90 for their conditions (survive or death) and an EQ-5D quality of life score.

### Sample size calculation

The predicted sample size is calculated to achieve the primary outcome, which is evaluating the diagnostic test accuracy of the ddPCR method (our index test) compared with the reference test.

According to previous published studies evaluating diagnostic accuracy of ddPCR in patients with suspected sepsis, expected test sensitivity (SN) and specificity compared with blood culture are 85% and 75%, respectively according to our pre-test and the previous studies [11, 14]. And the estimated disease prevalence (P), which is the blood culture positive rate in this case, is around 3% to 15% [5, 21, 22]. Therefore, based on the sample size calculation statistical methodology incorporating in the prevalence of disease [23, 24], with a significance ($\alpha$) level of 0.05, a 95%CI width of 10% and a prevalence of 3%, the sample size for the experimental group is:

$$\mathrm{N}1=\frac{TN+FN}{P}= {Z}\alpha^2/2\frac{SN(1-SN)}{{W}^{2}*P}=1632.$$

Since patients will be allocated into two groups with a ratio of 3:1, the total sample size of the two groups combined is 1632/3*4 = 2176. Assuming a 5% drop-out rate in follow-up, the sample size is calculated to be 2292 patients.

### Outcome measures

The primary outcome of this trial is the diagnostic efficacy of the ddPCR assay (the index test). The estimated sensitivity and specificity will be determined by comparing ddPCR assay results with the synchronous blood culture (the reference test) results from the prospective clinical specimens. Secondary outcomes between two arms consist of clinical outcomes and surrogate endpoints and fall into four categories: ① Clinical indicators: mortality rates at follow-up time points until day 90, the mean Sequential Organ Failure Assessment (SOFA) score at follow-up time points, the length of stay in the hospital or in the ICU, time to remission from shock (if there is septic shock), duration of mechanical ventilation or vasoactive drug application, rates of new requirement for renal replacement therapy;② Microbial and antibiotic usage indicators: time from enrollment to identification of a pathogen or the directed antimicrobial therapy, duration of major broad-spectrum antibiotics(glycopeptide, carbapenem) use, duration of antibiotic use;③ Quality of life indicator: the EQ-5D-5L score on day 90, which is a standardized measure of health-related quality of life developed by the EuroQol Group comprising five dimensions: mobility, self-care, usual activities, pain and discomfort, and anxiety and depression.④ Health economic indicators: total cost during hospital stay.

### Statistical methods

Continuous general characteristics and laboratory test variants will be described by medians when they do not conform to the Kolmogorov–Smirnov test and by means when do. The Chi-square test will be used to evaluate independent binomial variables. All hypothesis tests are two-sided except for the primary outcome and a p value < 0.05 is considered of significance. For the primary outcome, the paired results of the ddPCR assay and BC will be analyzed using McNemar's test. Wilcoxon rank-sum test will be used to compare efficacy grades, Fisher's exact test to compare effective rates, and CMH (Cochran-Mantel–Haenszel) method to compare the efficacy grade and response rate after deducting the central effect. Multivariate analysis of multivariate mixed effects logistic regression can be used for outcome variables, and multivariate analysis of variance-adjusted Cox models can be used for time-event outcome variables. Apart from multivariate analysis, and propensity score methods may be used to control bias. In this trial, there will be an intention-to-treat (ITT) analysis as randomized analysis, including that of discontinuation and deviation. Also, sensitivity analysis will be used to ensure appropriate interpretation. Methods like multiple imputation will be used to handle missing data. Figures and statistical analyses will be conducted using the Prism 9.0 software (GraphPad Software, San Diego, CA, the USA), SPSS Version 20.0 (IBM Corp., Armonk, NY, USA), and R studio 4.0.3 software (R studio Software, Boston, MA, USA).

### Data collection and quality management

Several measures should be established and practiced to ensure the sound process before and during project implementation. All the personnel involved in the project, including clinicians, laboratory, and project management personnel, are well trained. Moreover, the involved clinicians have been specially trained so that they are familiar with the inclusion and exclusion criteria of this research and relevant topics. Besides, to ensure the cases recruited are of good quality and representative, once the case meets the inclusion standards, it must be included rather than be selected or omitted artificially. Furthermore, specialized personnel in charge of Huashan Hospital will thoroughly review and assess every case. Clinical data of enrolled patients will require collection through a uniformly designed case report form (CRF).

In order to assure the data is of good quality and is collected accurately, physicians who are responsible for filling the clinical information have received standardized training. Therefore, every person in charge will have a clear understanding and cognition of the questions in the questionnaires when they record information or ask the participants face to face. The clinical data should be obtained in practice or acquired bedside. Additionally, the CRF returned will be well checked by a dedicated person to ensure that there are no missing items or other obvious mistakes. Furthermore, all relevant forms, such as registration books and CRFs, should be appropriately stored in a suitable place that is safe from natural or artificial damage. Therefore, the authenticity and objectivity of the subject material will be guaranteed, and the person should also ensure all the information can be accessed after the on-site investigation. Lost to follow-up, discontinuation and deviation will be reported and recorded. Physicians will try to obtain the missing data.

In addition, each center should conduct the research strictly according to the designed regulations to ensure the data collected is credible and the project is carried out correctly. Thus, supervisors will be assigned by the Huashan project team and the main researcher of each center will be supervised by the Huashan PROGRESS project team every two months. A contract research organization will take responsibility for the data monitoring and quality control twice a year, supervised by the Huashan PROGRESS project team. And independent data and safety monitoring committee is not needed for the low risk of this trial.

An interim analysis will be performed on the primary outcome when 50% of patients have been enrolled and have completed the day 90 follow-up. It will be performed by the Huashan PROGRESS project team who has full access to these interim results. Decision on the continuation of the trial will be reported to the Huashan ethics committee.

### Adverse event management

Potential adverse events have been evaluated. Patients may experience little psychological discomfort while collecting clinical medical history. In addition, in the research of this project, except for routine clinical testing, 10 ml extra peripheral blood will be drawn, and there will be no other examination, operation or cost required. However, the extra blood draw may cause short-term pain or bruise at the blood draw site. Some patients may even feel slightly dizzy. On infrequent occasions, needle site infections may occur.

All the adverse events will be recorded within two hours and subsequently evaluated by the research group. To manage and avoid adverse events, researchers will provide timely comfort if a participant has any psychological discomfort during the information collection. Besides, once the adverse event happens, the clinicians in charge will be informed immediately to make sure the patient will receive appropriate treatment. In an unexpected injury, the patient will receive appropriate treatment or compensation. All the adverse events in this research will be detailed recorded by a dedicated person. Severe adverse events will be timely recorded and reported to the ethics committee and relevant responsible departments.

### Confidentiality

On the basis of applicable privacy and confidentiality laws, participants’ personal information is only restricted to that necessary for outcome evaluation of study regimen and patients will not be identified through publicly available information. Paper documents containing participants’ information will be saved in a dedicated office in cooperative hospitals for at least 10 years. Digital documents will be kept in password-protected files on website so that study documents can only be accessed by authorized personnel.

## Discussion

Previous studies have confirmed that early diagnosis followed by timely prescription of appropriate antimicrobial therapy is associated with better outcomes of sepsis patients [3, 25]. Yet challenges remain for the etiology diagnosis. Since it is anticipated that rapid and accurate recognition of causative pathogens in the sepsis patients will be of great utility for their clinical outcomes, given the guidelines’ recommendation of empirical use of broad-spectrum antibiotics, many different biomarkers and mRDTs have been investigated to improve the etiology diagnosis.

However, most trials evaluating the new tests’ diagnostic accuracy are retrospective or prospective cohort study at present. A small number of RCTs provide data about the real-world performance of these mRDTs, especially those reporting the impact of interventions like mortality [10]. For the example, in the EMICA trial, a multi-center, open-label, cluster-randomized crossover trial of a total of 1416 patients with severe sepsis, the microbiological diagnosis rate was higher during intervention period (additional testing with LightCycler^®^SeptiFast than during control period [42.6% (198/465) vs. 28.1% (125/442), the turn-around time was shorter (22.9 vs. 49.5 h) and hospital costs were similar [26]. Several previous studies have reported the diagnostic accuracy of the ddPCR based assays. Wousters et al. [11] applied ddPCR to 45 blood samples collected from patients with suspected BSI, and the overall sensitivity and specificity was 80% (95% CI 0.52–0.96) and 87% (95% CI 0.69–0.96) compared with blood culture, respectively. Zhou et al. [27] evaluated the ddPCR detection method using pleural and peritoneal fluid samples, and the sensitivity was 96% (95% CI = 79.65–99.90%) and 92.86% (95% CI = 66.13–99.82%), respectively. Furthermore, Hu et al. [14] performed a head-to-head comparison between the ddPCR assay and metagenomic NGS in 45 critically ill patients. They found that for pathogens within the target range, the ddPCR assay showed a higher detection rate than mNGS (the found 88 positives in ddPCR and only 53 positives in mNGS), while mNGS (n = 126) detected more species of DNA pathogens than that detected by ddPCR (88 in ddPCR and 126 in mNGS). However, most of the studies focused on diagnostic test accuracy. PROGRESS trial is the first pragmatic RCT to explore the clinical impact of a rapid ddPCR assay in patients with suspected sepsis. Our study can make up one of the limitations of many previous studies, that the change in antimicrobial therapy were analyzed post-hoc leading to mal-interpreting, instead of during the actual clinical course. Also, our randomized controlled trial has the ability to reduce confounding of the intervention, and it can evaluate whether therapeutic adjustment as a result of ddPCR results will have an impact on the clinical outcomes.

The PROGRESS trial is a comparative effectiveness research (CER) for the purpose of evaluating an alternative health-care intervention, which, in this case, is the appliance of a rapid ddPCR diagnostic assay. It can also be classified as a practical RCT (pRCT), and the pragmatic approaches are taken in many aspects. Firstly, the inclusion criteria are designed to enroll any adult patient with suspected sepsis who is likely to be provided the intervention, including people who are immune compromised, with multiple morbidities, or over the age of eighty. Secondly, to set the situation as consistent as possible with the real-world condition, we do not interfere with other pathogen tests that clinicians may choose. Microbial examinations like mNGS are accessible to both the experiment group and the control group. Thirdly, except for day 90 follow-up on the phone, the follow-up will be completed during the patients’ hospital stay. Lastly there will be an intention-to-treat analysis using all available data. It not only maintains a relatively high internal authenticity through the randomized controlled trial design, but also takes into account generalizability and feasibility in ‘the real world’ when assessing the benefits of the ddPCR assay [28].

There are also several limitations about this trial. One limitation is that when calculating turnaround time, we assume the availability of the ddPCR assay is equal to that of standard blood culture, and it is not true. The ddPCR assay hasn’t been integrated into the usual clinical procedures. It is only available for twelve hours during the daytime on weekdays because of the limited number of trained staff to run the ddPCR test at this day. We believe next steps for such a novel mRDT will be making the process of handling samples and interpreting results easier to operate, as well as minimizing the need for technical expertise. And we will add the waiting time into the turnaround time in the final analysis when comparing with that of the blood culture. Also, the assay only includes the fifteen most common detected pathogens in BSIs. It cannot investigate unknown emerging pathogens, and the ddPCR panel’s ability to expand is to be studied. Moreover, since it is not a double-blind RCT, bias such as clinical decision-making preferences are inevitable. And as a comparative effectiveness research and a real-world study, statistical methods need to be taken to further control the confounding factors.

## Data Availability

This is a study protocol manuscript, not applicable.

## Abbreviations

pRCT: Pragmatic randomized controlled trial
SIRS: System inflammatory reaction syndrome
mRDTs: Molecular rapid diagnosis tests
mNGS: Metagenomic next-generation sequencing
RWS: Real-world study
BSIs: Blood stream infections
TAT: Turnaround time
CRF: Case report form
PCR: Polymerase chain reaction
CCI score: Charlson Comorbidity Index score
SOFA score: Sequential Organ Failure Assessment score
